# Clinical symptoms, thyroid dysfunction, and metabolic disturbances in first-episode drug-naïve major depressive disorder patients with suicide attempts: A network perspective

**DOI:** 10.3389/fendo.2023.1136806

**Published:** 2023-02-24

**Authors:** Pu Peng, Qianjin Wang, Xiaoe Lang, Tieqiao Liu, Xiang-Yang Zhang

**Affiliations:** ^1^Department of Psychiatry, and National Clinical Research Center for Mental Disorders, The Second Xiangya Hospital of Central South University, Changsha, Hunan, China; ^2^Department of Psychiatry, First Hospital of Shanxi Medical University, Taiyuan, China; ^3^Chinese Academy of Science (CAS) Key Laboratory of Mental Health, Institute of Psychology, Chinese Academy of Sciences, Beijing, China; ^4^Department of Psychology, University of Chinese Academy of Sciences, Beijing, China

**Keywords:** suicidal attempt, major depressive disorder, network analysis, thyroid stimulating hormone, metabolic disturbances

## Abstract

**Backgrounds:**

Co-occurrence of thyroid dysfunction, metabolic disturbances, and worsening clinical symptoms in major depressive disorder (MDD) patients with suicidal attempts (SA) are common. However, their relationship in SA patients remains unexplored. We aimed to (1) determine the independent association of thyroid dysfunction, clinical symptoms, and metabolic disturbances with SA; and (2) identify their interactions in SA patients *via* the network approach.

**Methods:**

1718 FEDN MDD patients were recruited. Depressive, anxiety, and psychotic symptoms were assessed by the Hamilton Rating Scale for Depression (HAMD), the Hamilton Rating Scale for Anxiety (HAMA), and the Positive and Negative Syndrome Subscale positive subscale, respectively. The serum levels of thyroid hormones and other metabolic parameters were assessed. Logistic regression model was applied to determine the correlates of SA. Network analysis was applied to determine the interaction between thyroid dysfunction, clinical symptoms, and metabolic disturbances.

**Results:**

SA patients had significant worse metabolic disturbances, thyroid dysfunction, and clinical symptoms than non-SA patients. Thyroid peroxidases antibody, thyroid stimulating hormone (TSH), HAMD scores, HAMA scores, and systolic blood pressure was independently associated with SA. Network analysis suggested that TSH was the hub of the network, exhibiting substantial associations with metabolic disturbances, anxiety, and psychotic symptoms in SA patients.

**Conclusions:**

Our work highlights the predominant role of serum TSH levels in the pathophysiology of SA. Regular thyroid function tests might help early detect SA. Targeting increased TSH levels may help reduce metabolic disturbances and clinical symptoms in SA patients.

## Introduction

Suicide is the most devastating consequence of patients with major depressive disorder (MDD). The lifetime prevalence of suicidal ideation, suicidal planning, and suicidal attempts (SA) in MDD patients is 37.7%, 15.1%, and 23.7%, respectively (1, 2). A recent meta-analysis shows that MDD patients are approximately 7 times more likely to have SA in the past year than healthy individuals (3). The high prevalence of SA highlights the strong need to identify the potential risk factors for SA, which is valuable for SA screening and intervening in MDD patients.

Although numerous studies have identified demographic and clinical risk factors for SA in MDD patients (4, 5), the biological correlates of SA remain largely unexplored (5). Several studies have suggested that thyroid hormones and metabolic parameters can be potential biomarkers for SA (6–9). However, the reported results are inconsistent. For example, one meta-analysis published in 2020 demonstrated that SA was associated with low serum levels of total cholesterol (TC) and low-density lipoprotein cholesterol (LDL-C) in 7068 patients with MDD (7). However, this view has been challenged by two recent large-scale studies (N=1279 and N=580) (10, 11), which found higher concentrations of TC and LDL-C in MDD patients with SA. Similarly, the relationship between thyroid dysfunction and SA in patients with MDD was also in debate. Several studies indicated that elevated TSH increased the risk of suicide (11), whilst some found an inverse association (12) or no association (13). The inconsistency in the previous studies may be due to the different study samples. Studies have shown that disease duration, comorbidities, and medication may have a substantial impact on thyroid function and metabolism (14–16), which may obscure their association with SA. Therefore, assessing the relationship between thyroid dysfunction, metabolic disturbances, and SA in first-episode drug-naïve (FEND) MDD patients may provide more solid evidence.

Studies have confirmed that the co-occurrence of thyroid dysfunction and metabolic disturbances is very common (17–20), especially in patients with MDD (21). For example, Kim et al. found that subclinical hypothyroidism increased the risk of metabolic syndrome by 7 times among individuals with depression (21). Two recent studies also found that MDD patients with SA exhibited more severe clinical symptoms, metabolic disturbances, and thyroid dysfunction than those without (11, 22). However, no prior study directly evaluated their relationship in patients with SA. Clarifying whether and how thyroid dysfunction, metabolic disorders, and clinical symptoms are interconnected in patients with SA may provide new insights into the pathophysiology of SA.

Network analysis, as an emerging tool, has advantages over traditional methods such as regression models in visualizing and describing independent associations between variables (23, 24). In a network model, a variable is visualized as a “node”. After sufficient adjustment for other variables within the network, the unique association between two variables is visualized as an “edge”. In addition to identifying correlations between variables, network analysis identifies the most influential variables that are most closely linked to the other variables in the network (i.e., central variables). The central variable is considered to play an important role in triggering and maintaining the network (25). Hence, the central variable may be a promising target for clinical interventions to reduce thyroid dysfunction, metabolic disturbances, and clinical symptoms in MDD patients with SA.

To date, emerging studies have applied network analysis to assess associations between variables in clinical medicine (26, 27). For example, Jia et al. have assessed the association of lipid markers with cognition performance and depression through a network approach (27). A recent study also determined the networks of lipid metabolism, inflammation, and depressive symptoms (26). However, there are no previous studies evaluating the network of clinical symptoms, thyroid dysfunction, and metabolic disturbance in MDD patients with SA, which gave us the motivation to conduct the present study. We recruited a large sample of FEDN MDD patients and evaluated SA, metabolic parameters, thyroid hormones, and clinical symptoms. We have two main aims (1): to determine the association of SA with clinical symptoms, metabolic disturbances, and thyroid dysfunction in first-episode drug-naïve patients with MDD; and (2) to determine the inter-relationship between metabolic disturbances, thyroid dysfunction, and clinical symptoms in patients with SA *via* the network approach.

## Methods

### Study procedure and participants

Participants were recruited at the psychiatric outpatient department of the First Hospital of Shanxi Medical University from 2015 to 2017. Inclusion criteria were as follows: (1) fulfilling DSM-IV criteria for MDD, diagnosed by two trained psychiatrists using the Structured Clinical Interview for DSM-IV Disorders (SCID); (2) 17-item Hamilton Depression Scale (HAMD) score of more than 23; (3) age 18-60 years old, Han nationality; (4) no prior medication, including antidepressant, antipsychotic drugs, thyroid hormone therapy, hypoglycemic agents, antihypertensive and lipid-lowering drugs; and (5) depression symptoms were first-episode and the disease duration of no more than 24 months. Exclusion criteria included: (1) pregnant or breastfeeding women; (2) concurrent DSM-IV axis I disorder including bipolar disorder, schizophrenia, and schizoaffective or severe medical conditions; (3) substance use disorder except for tobacco; and (4) unwillingness to provide informed consent.

All participants provided written informed consent. This study was approved by the Institutional Review Board (IRB) of the First Hospital of Shanxi Medical University (No. 2016-Y27).

### Interview and clinical assessments

We collected basic information, including age, gender, education, onset, and duration of MDD, and married status through a self-designed questionnaire. All participants were independently interviewed face-to-face by two trained psychiatrists *via* the SCID. Two psychiatrists independently assessed each participant’s depression, anxiety, and psychotic symptoms by the HAMD, Hamilton Anxiety Scale (HAMA), and the positive subscale of Positive and Negative Syndrome Subscale (PANSS), respectively. HAMD score ranges from 0-52, with a cutoff point of 24 being used to determine severe depression (28). HAMA consists of 14 items, measuring psychological and somatic anxiety symptoms (29). It applied the 5-Likert scale, with a total score ranging from 0-56. The PANSS positive subscale assesses seven positive symptoms (30). The PANSS-positive subscale score ranges from 7-49. Higher scores on the HAMA, HAMD, and PANSS indicate more severe symptoms. These three scales have been validated and widely used in the Chinese population (31–33). According to previous studies (34, 35), HAMA score >20 and PANSS positive subscale score >14 indicate significant anxiety and psychotic symptoms, respectively. The correlation coefficients between the two psychiatrists’ scores on all three scales were higher than 0.8.

We assessed SA through face-face interviews. All participants were asked the question: “In your lifetime, did you ever try to kill yourself?”. This single item has been validated and used widely in previous epidemiological studies for the detection of SA (36, 37). Those who answered “yes” were considered to have lifetime SA. We further asked them about the timing and frequency of SA. We contacted the family members of the participants for the details of SA when patients were unable to provide definitive information.

### Biochemical indicators

Blood samples were collected in the morning after an overnight fast before participants received any medical treatment. Serum levels of free triiodothyronine (FT3), free thyroxine (FT4), thyroid stimulating hormone (TSH), antithyroglobulin (TgAb), thyroid peroxidase antibody (TPOAb), TC, TG, high-density lipoprotein (HDL-C), low-density lipoprotein (LDL-C), and glucose were assessed. Lipid markers (TC, TG, HDL-C, LDL-C) and glucose were measured on a Cobas E610 (Roche, Basel, Switzerland). Thyroid hormones were assayed on a Roche C6000 Electrochemiluminescence Immunoassay Analyzer (Roche Diagnostics, Indianapolis, IN, USA). Measurements were conducted in the laboratory of the First Hospital, Shanxi Medical University. The nurses measured the patients’ weight, height, and blood pressure. We calculated body mass index (BMI) according to the following formula: BMI = Weight (kg)/Height (m) ^2^.

According to previous studies in the Chinese population (38, 39), metabolic disturbances and thyroid dysfunction were defined as follows: (1) overweight or obesity: BMI≥24; (2) hyperglycemia: glucose≥6.1mmol/L; (3) hypertension: SBP≥140 mmHg and/or DBP≥90mmHg; (4) hypertriglyceridemia: TG≥2.3 mmol/L; (5) low HDL: HDL-C ≤ 1.0 mmol/L; (6) hypercholesterolemia: TC≥6.2 mmol/L or LDL-C≥4.1 mmol/L; (7)abnormal TgAb: TgAb≥115 IU/L; (8) abnormal TPOAb: TPOAb ≥34 IU/L; (9) subclinical hypothyroidism (SCH): TSH >4.2 mIU/L with normal fT4 concentration (10–23 pmol/L); (10) hyperthyroidism: TSH<0.27 mIU/L and FT4 >23 pmol/L, and (11) hypothyroidism: TSH >4.2 mIU/L with low FT4 concentration (<10 pmol/L).

### Statistical analysis

#### Data processing

According to the Shapiro-Wilk test, the continuous data in our study were not normally distributed. Therefore, we expressed the continuous data as the median and interquartile range (IRQ; 25-75%) and the categorical data as frequencies and percentages. All statistical analyses were conducted on R (ver. 4.20). We adopt two-tailed tests with p<0.05 indicating statistical significance.

#### Univariate and multiple analyses

We assessed differences in metabolic disturbances, clinical symptoms, and thyroid dysfunction between MDD patients with and without SA by chi-square test, Fisher’s exact test, and Whitney U test, as appropriate. Bonferroni correction was employed for multiple testing (p’=0.05/40 = 0.00125). A multiple logistic regression model was conducted to identify independent correlates of SA. Variables with P < 0.05 in univariate tests were included in the multiple logistic regression analysis using the stepwise method.

#### Network analysis

Clinical symptoms (HAMA, HAMD, and PANSS scores), metabolic parameters (TC, TG, LDL-C, HDL-C, SBP, DBP, and BMI), and thyroid hormones (TSH, TPOAb, TgAb, FT3, and FT4) were included in the network. Following a previous study (40), we performed nonparanormal transformations using Rpackage “huge” because the data were not normally distributed. We estimated and visualized the network using Rpackage “qgraph” and “bootnet” (41). We estimated the network using the default of the EBICglasso model, which was widely used in psychological network models (42).γ was set to 0.5, which made the network more sparse and strikes a balance between sensitivity and specificity in preserving true edges. The network consisted of “nodes” (i.e., metabolic parameters, thyroid function, and clinical symptoms) and “edges” (i.e., pairwise correlations between two nodes after controlling for other variables within the network). Thicker edges implied a greater association (43). Red edges indicated negative associations, while blue edges indicated positive associations. We calculated the centrality index “strength” to quantify the importance of the nodes. Nodes with higher strength were considered to exhibit strong associations and impacts on other nodes within the network. We also calculated the predictability of the nodes by Rpackage “MGM” (44). Similar to the R^2^ in the regression model, predictability referred to the extent to which the variance of a node can be explained by other nodes in the network (45).

Finally, we evaluated the stability and accuracy of our network by Rpackage “bootnet”. Bootstrap procedures were performed with 1000 bootstrap samples to determine the accuracy of the estimated edges. We conducted a case-dropping procedure to evaluate the stability of the network. The correlation stability coefficient (CS-C) was calculated, and a CS-C above 0.5 implied reasonable stability.

## Results

### Sample characteristics

We recruited 1718 FEDN MDD patients (**Table 1**). The majority of the participants were female (1130, 66%), married (1216, 71%), and had a degree below college (1173, 68%). One-fifth of the participants (346, 20%) had lifetime SA. 235 (14%) had SA in the past two weeks.

**Table 1:** Sample characteristics of SA and non-SA patients.

Variable	Overall, N = 1,718^1^	Without SA, N = 1,372^1^	With SA, N = 346^1^	p-value^2^
**Age, year**	34 (23, 45)	33 (23, 45)	35 (25, 47)	0.023
**Duration, month**	5 (3, 8)	5 (3, 8)	6 (3, 9)	<0.001
**Onset, year**	34 (23, 45)	33 (23, 45)	34 (25, 47)	0.026
**Gender**				0.4
Male	588 (34%)	476 (35%)	112 (32%)	
Female	1,130 (66%)	896 (65%)	234 (68%)	
**Education**				0.5
Below college	1,173 (68%)	932 (68%)	241 (70%)	
College or above	545 (32%)	440 (32%)	105 (30%)	
**Married**	1,216 (71%)	965 (70%)	251 (73%)	0.4
**PANSS**	7 (7, 7.8)	7 (7, 7)	8 (7, 17.8)	<0.001
**Psychotic symptom**	171 (10.0%)	83 (6.0%)	88 (25%)	<0.001
**HAMD**	30 (28, 32)	30 (28, 32)	32 (30, 34)	<0.001
**HAMA**	21.0 (18.0, 23.0)	20.0 (18.0, 22.0)	23.0 (21.0, 26.0)	<0.001
**Anxiety**	894 (52%)	610 (44%)	284 (82%)	<0.001
**TSH, uIU/L**	4.91 (3.11, 6.66)	4.63 (2.89, 6.14)	6.76 (4.54, 8.89)	<0.001
**TgAb, IU/L**	21 (14, 44)	20 (14, 32)	28 (18, 144)	<0.001
**TPOAb, IU/L**	17 (12, 35)	16 (12, 29)	29 (14, 171)	<0.001
**FT3, pmol/L**	4.92 (4.38, 5.41)	4.91 (4.39, 5.40)	4.92 (4.34, 5.44)	>0.9
**FT4, pmol/L**	16.5 (14.4, 18.7)	16.5 (14.4, 18.8)	16.5 (14.4, 18.6)	0.9
**Glucose, mmol/L**	5.34 (4.94, 5.80)	5.28 (4.92, 5.71)	5.56 (5.05, 6.10)	<0.001
**TC, mmol/L**	5.22 (4.46, 6.00)	5.11 (4.36, 5.81)	5.72 (4.95, 6.59)	<0.001
**HDLC, mmol/L**	1.23 (1.01, 1.42)	1.25 (1.05, 1.44)	1.13 (0.89, 1.30)	<0.001
**TG, mmol/L**	1.97 (1.40, 2.77)	1.94 (1.37, 2.74)	2.16 (1.46, 2.93)	0.004
**LDLC, mmol/L**	2.96 (2.38, 3.52)	2.90 (2.30, 3.42)	3.21 (2.60, 3.74)	<0.001
**BMI, kg/m2**	24.23 (23.22, 25.60)	24.23 (23.23, 25.60)	24.27 (23.18, 25.99)	0.8
**SBP, mmHg**	120 (112, 127)	120 (111, 126)	125 (116, 134)	<0.001
**DBP, mmHg**	76 (70, 80)	75 (70, 80)	78 (74, 84)	<0.001
**Abnormal TgAb**	297 (17%)	191 (14%)	106 (31%)	<0.001
**Abnormal TPOAb**	438 (25%)	282 (21%)	156 (45%)	<0.001
**SCH**	1,041 (61%)	778 (57%)	263 (76%)	<0.001
**Hyperthyroidism**	5 (0.3%)	5 (0.4%)	0 (0%)	0.6
**Hypothyroidism**	3 (0.2%)	2 (0.1%)	1 (0.3%)	0.5
**Hyperglycemia**	241 (14%)	153 (11%)	88 (25%)	<0.001
**Low HDL**	429 (25%)	306 (22%)	123 (36%)	<0.001
**Overweight or obesity**	1,026 (60%)	825 (60%)	201 (58%)	0.5
**High SBP**	53 (3.1%)	16 (1.2%)	37 (11%)	<0.001
**High DBP**	74 (4.3%)	38 (2.8%)	36 (10%)	<0.001
**Hypertriglyceridemia**	668 (39%)	512 (37%)	156 (45%)	0.008
**Abnormal TC**	357 (21%)	225 (16%)	132 (38%)	<0.001
**Abnormal LDL-C**	185 (11%)	125 (9.1%)	60 (17%)	<0.001
**Hypertension**	92 (5.4%)	42 (3.1%)	50 (14%)	<0.001
**Hypercholesterolemia**	421 (25%)	277 (20%)	144 (42%)	<0.001

SCH, subclinical hypothyroidism; HAMD, Hamilton Depression Rating Scale; HAMA, Hamilton Anxiety Rating Scale; PANSS, the Positive and Negative Syndrome Scale; TSH, thyroid-stimulating hormone; FT3, free triiodothyronine; FT4, free thyroxine; TgAb, antithyroglobulin; TPOAb, thyroid peroxidases antibody; TC, total cholesterol; HDL-C, high-density lipoprotein; LDL-C, low-density lipoprotein; TG, total triglycerides; BMI, body mass index.

^1^Median (IQR); n (%)

^2^Wilcoxon rank sum test; Pearson’s Chi-squared test.

### The difference in metabolic disturbances, thyroid function, and clinical symptoms in FEDN MDD patients with and without SA

SA patients tended to be older, had a longer duration of disease, and had a later onset (**Table 1**). Compared with non-SA patients, SA patients had significantly more severe metabolic disturbances, thyroid dysfunction, and psychological distress than non-SA patients, showing higher scores on HAMD, HAMA, and PANSS positive subscale. The prevalence rates of SCH, abnormal TgAb, abnormal TPOAb, hyperglycemia, abnormal TC, abnormal LDL-C, low HDL, hypertension, and hypercholesterolemia were significantly higher in SA patients than in non-SA patients. Their associations remained significant after the Bonferroni correction. In addition, SA patients were also more likely to have hypertriglyceridemia. However, the association between hypertriglyceridemia and SA was no longer significant after multiple testing.

### Independent correlates of SA in FEDN MDD patients

We conducted a multiple logistic regression model in variables showing p<0.05 in univariate analysis (i.e., age, duration, and the onset of MDD, HAMD, HAMA, PANSS, TSH, TPOAb, TgAb, TC, TG, HDL-C, LDL-C, glucose, SBP, and DBP). **Table 2** summarizes the results of the logistic regression model. HAMD (Odds ratio, OR, 1.081, 95% confidence intervals, 95% CI, 1.016-1.151, p=0.014), HAMA (OR, 1.251, 95%CI, 1.189-1.316, p<0.001), TSH (OR, 1.115, 95%CI, 1.047-1.187, p=0.001), TPOAb (OR, 1.002, 95%CI, 1.001-1.003, p<0.001), and SBP (OR, 1.023, 95%CI, 1.008-1.038, p=0.002) were independently associated with SA in FEDN MDD patients.

**Table 2:** Regression model of SA in MDD patients.

Characteristic	OR^1^	95% CI^1^	p-value
**HAMD**	**1.081**	**1.016, 1.151**	**0.014**
**HAMA**	**1.251**	**1.189, 1.316**	**<0.001**
**TSH**	**1.115**	**1.047, 1.187**	**0.001**
**TPOAb**	**1.002**	**1.001, 1.003**	**<0.001**
**SBP**	**1.023**	**1.008, 1.038**	**0.002**

HAMA, Hamilton Anxiety Rating Scale; HAMD, Hamilton Depression Rating Scale; TSH, thyroid-stimulating hormone; TPOAb, thyroid peroxidase antibody; SBP, systolic blood pressure.

^1^: OR = Odds Ratio, CI = Confidence Interval

### Network of thyroid dysfunction, metabolic disturbances, and clinical symptoms in MDD patients with SA

**Figure 1:**
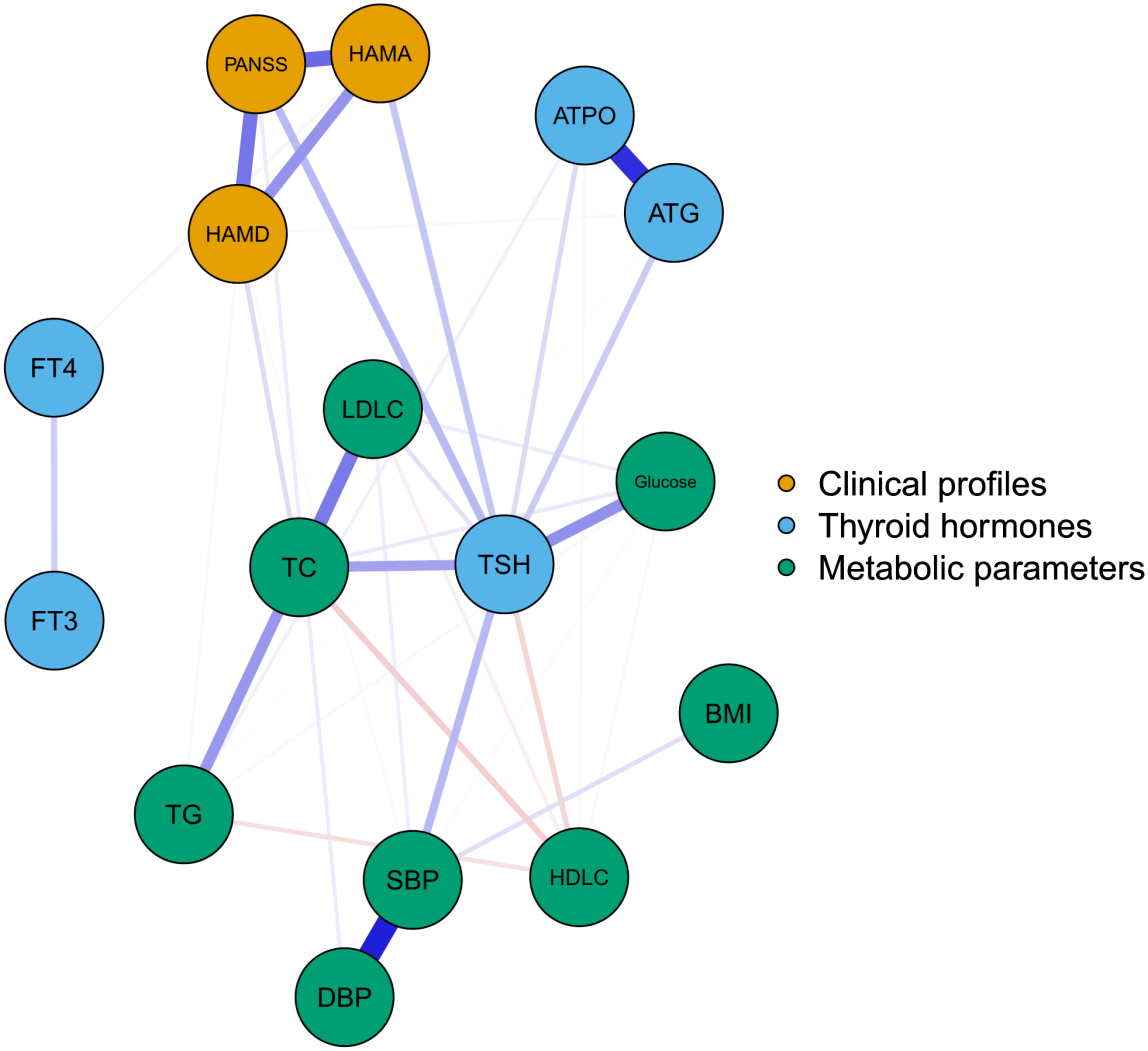
The network of thyroid-dysfunction, metabolic disturbances, and clinical symptoms in FEDN MDD patients with suicidal attempts. Blue, orange, and green nodes represented thyroid hormones, clinical symptoms, and metabolic parameters, respectively. Blue and red edges indicated positive and negative associations, respectively. Thicker edges suggested stronger associations. HAMD = Hamilton Depression Rating Scale, HAMA = Hamilton Anxiety Rating Scale, PANSS = the Positive and Negative Syndrome Scale, TSH = thyroid-stimulating hormone, FT3 = free triiodothyronine, FT4 = free thyroxine, TgAb = antithyroglobulin, TPOAb = thyroid peroxidases antibody, TC = total cholesterol, HDL-C = high-density lipoprotein, LDL-C = low-density lipoprotein, TG = total triglycerides, BMI = body mass index.

**Figure 1** illustrates the network of thyroid dysfunction, metabolic disturbances, and clinical profiles in MDD patients with SA. The network was composed of 16 nodes and 32 edges. Visually, TSH was in the center of the network. It exhibited a strong positive association with metabolic parameters including SBP, TC, and glucose. TSH was also positively correlated with PANSS and HAMA. In contrast, BMI, FT3, and FT4 were at the margin of the network, exhibiting a very weak association with clinical symptoms. We also observed a strong association between PANSS, HAMA, and HAMD. The correlation matrix between the nodes is presented in **Table S1**.

**Figure 2:**
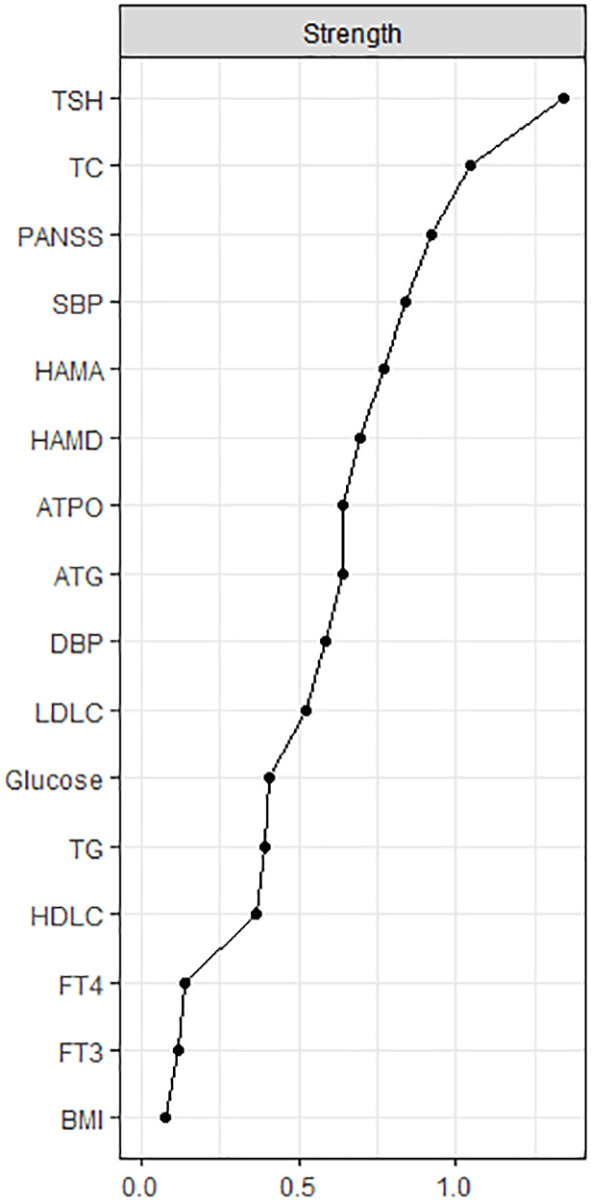
The centrality plot of the network. The X-rays represented the strength of each node. Nodes with higher strength have stronger impact in other nodes within the network. HAMD = Hamilton Depression Rating Scale, HAMA = Hamilton Anxiety Rating Scale, PANSS = the Positive and Negative Syndrome Scale, TSH = thyroid-stimulating hormone, FT3 = free triiodothyronine, FT4 = free thyroxine, TgAb = antithyroglobulin, TPOAb = thyroid peroxidases antibody, TC = total cholesterol, HDL-C = high-density lipoprotein, LDL-C = low-density lipoprotein, TG = total triglycerides, BMI = body mass index.

The centrality plot (**Figure 2**) confirmed that TSH was the central node of the network, followed by TC and PANSS scores. **Table S2** displays the predictability of the nodes in the network. The predictability of TSH was the highest (0.57). The predictability of clinical symptoms was 0.53 for PANSS, 0.50 for HAMA, and 0.45 for HAMD. These results indicated that half of the variance of clinical symptoms could be explained by the nodes in the network. The lowest predictability was found for FT3, FT4, and BMI.

The network had reasonable stability with a value of 0.671 for CS-C (**Figure S1**), indicating that after omitting 67% of the raw data, the network remained highly correlated with the original network (r=0.7). The bootstrap procedure also demonstrated high accuracy of the estimated edges within the network (**Figure S2**).

## Discussion

To our knowledge, this is the first study to explore the relationship between thyroid dysfunction, metabolic disturbances, and clinical symptoms in SA patients through a network approach. Our main findings included (1): SA MDD patients exhibited more severe metabolic disturbances, thyroid dysfunction, and clinical symptoms compared to non-SA MDD patients; (2) the severity of anxiety and depression symptoms, SBP, TSH, and TPOAb were independently associated with SA in FEDN MDD patients; and (3) TSH played an important role in the network of thyroid dysfunction, metabolic disturbances, and clinical symptoms in SA patients. Taken together, our work highlights the predominant role of serum TSH levels in the pathophysiology of SA. In addition to being a potential biomarker for SA in MDD patients, the serum TSH level is closely associated with SA-related metabolic disturbances and clinical symptoms. Hence, regular thyroid function tests might help early detect SA. Targeting increased TSH levels may help to reduce metabolic disturbances and clinical symptoms in MDD patients with SA.

Consistent with previous studies (46–48), our study demonstrated a very high metabolic burden and thyroid dysfunction in patients with SA, which called for regular metabolic and thyroid function tests in this population. There are a few explanations for the biological changes in SA patients. First, SA patients have more severe depressive symptoms, which may lead to an unhealthy lifestyle, such as irregular sleep and diet, resulting in metabolic disturbances and thyroid dysfunction (49). Second, inflammation may act as a bridge between SA and metabolic disorders. Emerging studies have found that inflammation plays an important role in MDD and its associated SA (50–54). Metabolic disorders were found to be associated with a chronic inflammatory state (55) and therefore may contribute to SA. Third, thyroid dysfunction was tightly associated with abnormal neurotransmitters (e.g., 5-hydroxytryptamine and norepinephrine), which played an important role in SA (56). Fourth, the high level of TPOAb might indicate the autoimmune status of MDD patients with SA. The disturbances in the kynurenine pathway and hypothalamic-pituitary-adrenal axis in autoimmune status might contribute to the SA (57).

Network analysis suggested that thyroid dysfunction, metabolic disturbance, and clinical symptoms were highly correlated among SA patients. High TSH levels were found to be the central variables within the network, which were tightly associated with both metabolic disturbances (impaired glucose metabolism, lipid metabolism, and hypertension) and clinical symptoms (psychotic and anxiety symptoms) in SA patients. The strong association of TSH with metabolic disturbances replicates findings in the general population (17), which can be explained by the following points. First, serum TSH levels can regulate lipid metabolism in various ways (58). High TSH levels can regulate cholesterol metabolism by binding to TSH receptors on the surface of hepatocytes (59). It can accelerate cholesterol synthesis and reduce cholesterol clearance (58), which can lead to dyslipidemia and obesity. Second, TSH levels may also play a role in insulin resistance and glucose tolerance (60). Studies have shown that high TSH levels are associated with the impairment of glucose transport (61).

Emerging studies suggested that thyroid dysfunction could predict several negative consequences in patients with MDD, including long-term readmission, conversion to bipolar disorder, and anxiety (62–64). However, most of these association was observed in the context of overt hypothyroidism. The relationship between SCH and clinical symptoms in patients with MDD remained controversial. Meta-analysis suggested that SCH exhibited a rather weak association with depressive symptoms (65). One population-based study demonstrated a negative association of TSH levels with anxiety (66). Interestingly, Liu et al. reported the same results as ours (11), finding higher serum TSH levels were associated with anxiety and psychosis among 1279 patients with MDD. The different results may be due to differences in sample characteristics (MDD patients versus community samples). Unfortunately, the relationship between SCH and clinical symptoms in patients with MDD was mostly studied in the cross-sectional study. The biological mechanism remained largely unexplored. Further studies are needed to validate our findings and to assess the possible mechanisms.

Our study has several important clinical implications. First, our study showed a high prevalence of metabolic disorders, thyroid dysfunction, anxiety, and psychotic symptoms in MDD patients with SA. Therefore, screening for these problems is crucial in this particular population. Second, our study suggested the severity of anxiety and depression, TSH level, TPOAb level, and SBP were independently associated with SA. Regular monitoring of these clinical variables might help early detect and prevent SA. Third, our study highlighted the predominant role of TSH in the pathophysiology of SA. Targeting TSH may be valuable in reducing metabolic disorders, clinical symptoms, and thyroid dysfunction associated with SA. To date, a few studies have shown that thyroid hormone therapy is effective in improving lipid metabolism in patients with SCH (67). Some studies have also documented its effectiveness in the treatment of MDD and bipolar depression (68, 69), but the results are inconsistent (70, 71). Therefore, more studies are in need to test our hypothesis.

Our study has several limitations. First, we used a cross-sectional study design, which prevented us from drawing causal relationships. Second, this study is monocentric and includes only the Han Chinese population. It remains unknown whether our findings can be generalized to other populations. Third, we did not collect several important sociocultural risk factors for SA, such as stressful life events and economic hardship (5). In addition, we did not collect lifestyle factors, such as smoking and exercise, as well as diet, which are strongly associated with metabolic disturbances and thyroid dysfunction, and this should be remedied in future studies. Fourth, our study is mainly descriptive and the underlying biological mechanisms are unknown. Fifth, we assessed suicide attempts by a single item only. Application of a specific suicide rating scale may better assess various aspects of suicidality (suicidal ideation, suicide planning, and SA) and their relationship with clinical symptoms, metabolic disturbances, and thyroid dysfunction. Further longitudinal studies with a more comprehensive assessment of confounding factors and suicidality are needed to validate our findings.

In conclusion, our study demonstrates that MDD patients with SA have severe thyroid dysfunction, metabolic disturbances, and clinical symptoms. Anxiety, depression, TSH, TPOAb, and SBP were independently associated with SA in FEDN MDD patients. Targeting increased TSH in MDD patients with SA may help reduce metabolic disturbances, clinical symptoms, and thyroid dysfunction in SA patients.

## Data availability statement

The raw data supporting the conclusions of this article will be made available by the authors, without undue reservation.

## Ethics statement

All participants provided written informed consent. This study was approved by the Institutional Review Board (IRB) of the First Hospital of Shanxi Medical University (No. 2016-Y27). The patients/participants provided their written informed consent to participate in this study.

## Author contributions

PP, formal analysis, writing - original draft. QW and XL, writing – review and editing. TL and X-YZ, conceptualization, writing–review and editing. All authors contributed to the article and approved the submitted version.
