# Supplementary material for: Clinical symptoms, thyroid dysfunction, and metabolic disturbances in first-episode drug-naïve major depressive disorder patients with suicide attempts: A network perspective
Source: Front Endocrinol (Lausanne). 2023 Feb 24;14:1136806. doi: 10.3389/fendo.2023.1136806 (PMC9999007; doi:10.3389/fendo.2023.1136806)
Supplement: Supplementary file 1 [file DataSheet_1.docx]

Supplementary materials for “Clinical symptoms, thyroid dysfunction, and metabolic disturbances in first-episode drug-naïve major depressive disorder patients with suicide attempts: a network perspective”

Supplementary Table 1 The edges estimated in the network

Supplementary Table 2 The predictability of the nodes in the network

Supplementary Figure 1. The stability of the network according to the case-dropping procedure. The CS-C for the node strength was 0.671.

Supplementary Figure 2. The accuracy of the edges in the network

Supplementary Table 1 The edges estimated in the network

|  | PANSS | HAMD | HAMA | TSH | ATG | ATPO | FT3 | FT4 | Glucose | TC | HDLC | TG | LDLC | BMI | SBP | DBP |
| --- | --- | --- | --- | --- | --- | --- | --- | --- | --- | --- | --- | --- | --- | --- | --- | --- |
| PANSS | 0.000 | 0.331 | 0.359 | 0.168 | 0.000 | 0.000 | 0.000 | 0.000 | 0.000 | 0.000 | 0.000 | 0.014 | 0.000 | 0.000 | 0.001 | 0.048 |
| HAMD | 0.331 | 0.000 | 0.256 | 0.000 | 0.013 | 0.000 | 0.000 | 0.000 | 0.000 | 0.083 | 0.000 | 0.000 | 0.000 | 0.000 | 0.010 | 0.000 |
| HAMA | 0.359 | 0.256 | 0.000 | 0.136 | 0.000 | 0.000 | 0.000 | 0.020 | 0.000 | 0.000 | 0.000 | 0.000 | 0.000 | 0.000 | 0.000 | 0.000 |
| TSH | 0.168 | 0.000 | 0.136 | 0.000 | 0.124 | 0.085 | 0.000 | 0.000 | 0.266 | 0.223 | -0.098 | 0.000 | 0.072 | 0.000 | 0.170 | 0.000 |
| TgAb | 0.000 | 0.013 | 0.000 | 0.124 | 0.000 | 0.500 | 0.000 | 0.000 | 0.000 | 0.000 | 0.000 | 0.003 | 0.000 | 0.000 | 0.000 | 0.000 |
| TPOAb | 0.000 | 0.000 | 0.000 | 0.085 | 0.500 | 0.000 | 0.000 | 0.000 | 0.000 | 0.003 | -0.019 | 0.035 | 0.000 | 0.000 | 0.000 | 0.000 |
| FT3 | 0.000 | 0.000 | 0.000 | 0.000 | 0.000 | 0.000 | 0.000 | 0.118 | 0.000 | 0.000 | 0.000 | 0.000 | 0.000 | 0.000 | 0.000 | 0.000 |
| FT4 | 0.000 | 0.000 | 0.020 | 0.000 | 0.000 | 0.000 | 0.118 | 0.000 | 0.000 | 0.000 | 0.000 | 0.000 | 0.000 | 0.000 | 0.000 | 0.000 |
| Glucose | 0.000 | 0.000 | 0.000 | 0.266 | 0.000 | 0.000 | 0.000 | 0.000 | 0.000 | 0.053 | -0.018 | 0.014 | 0.047 | 0.000 | 0.006 | 0.000 |
| TC | 0.000 | 0.083 | 0.000 | 0.223 | 0.000 | 0.003 | 0.000 | 0.000 | 0.053 | 0.000 | -0.115 | 0.247 | 0.320 | 0.000 | 0.000 | 0.000 |
| HDLC | 0.000 | 0.000 | 0.000 | -0.098 | 0.000 | -0.019 | 0.000 | 0.000 | -0.018 | -0.115 | 0.000 | -0.076 | -0.041 | 0.000 | 0.000 | 0.000 |
| TG | 0.014 | 0.000 | 0.000 | 0.000 | 0.003 | 0.035 | 0.000 | 0.000 | 0.014 | 0.247 | -0.076 | 0.000 | 0.000 | 0.000 | 0.000 | 0.000 |
| LDLC | 0.000 | 0.000 | 0.000 | 0.072 | 0.000 | 0.000 | 0.000 | 0.000 | 0.047 | 0.320 | -0.041 | 0.000 | 0.000 | 0.000 | 0.042 | 0.000 |
| BMI | 0.000 | 0.000 | 0.000 | 0.000 | 0.000 | 0.000 | 0.000 | 0.000 | 0.000 | 0.000 | 0.000 | 0.000 | 0.000 | 0.000 | 0.076 | 0.000 |
| SBP | 0.001 | 0.010 | 0.000 | 0.170 | 0.000 | 0.000 | 0.000 | 0.000 | 0.006 | 0.000 | 0.000 | 0.000 | 0.042 | 0.076 | 0.000 | 0.538 |
| DBP | 0.048 | 0.000 | 0.000 | 0.000 | 0.000 | 0.000 | 0.000 | 0.000 | 0.000 | 0.000 | 0.000 | 0.000 | 0.000 | 0.000 | 0.538 | 0.000 |

Supplementary Table 2 The predictability of the nodes in the network

| Variable | Predictability |
| --- | --- |
| PANSS | 0.534 |
| HAMD | 0.452 |
| HAMA | 0.495 |
| TSH | 0.57 |
| TgAb | 0.41 |
| TPOAb | 0.399 |
| FT3 | 0.04 |
| FT4 | 0.033 |
| Glucose | 0.184 |
| TC | 0.483 |
| HDLC | 0.145 |
| TG | 0.166 |
| LDLC | 0.26 |
| BMI | 0.03 |
| SBP | 0.452 |
| DBP | 0.431 |

Supplementary Figure 1. The stability of the network


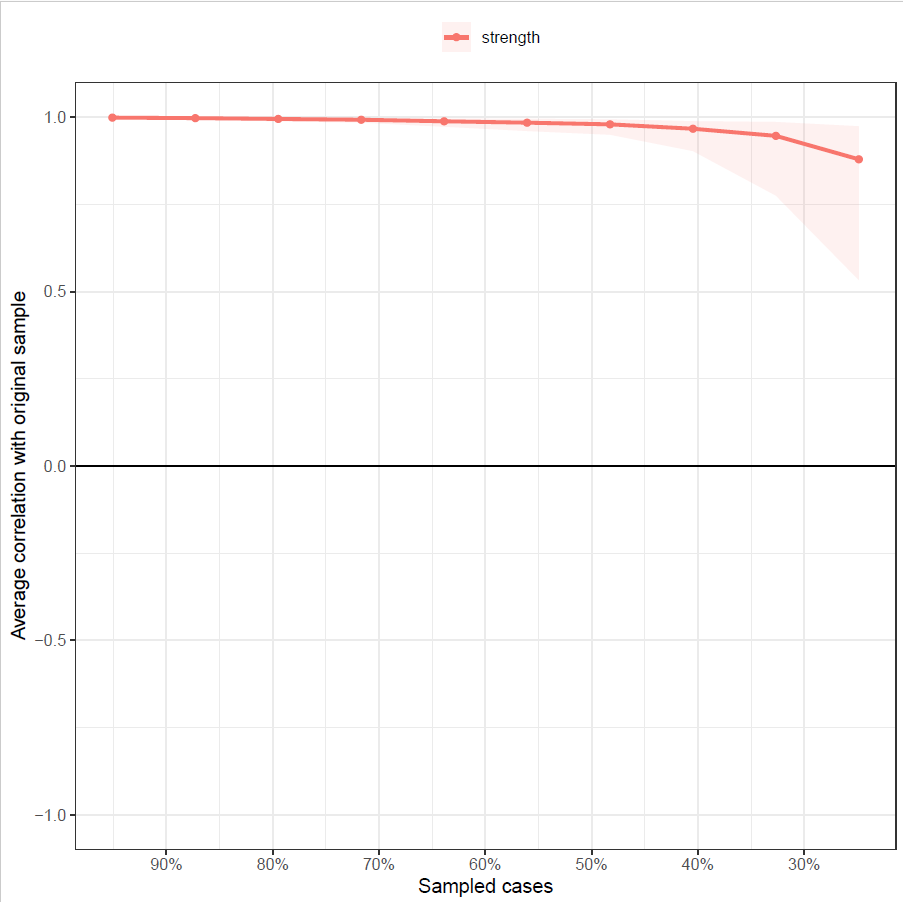


The stability of the network according to the case-dropping procedure. The CS-C for the node strength was 0.671.

Supplementary Figure 2. The accuracy of the edges in the network


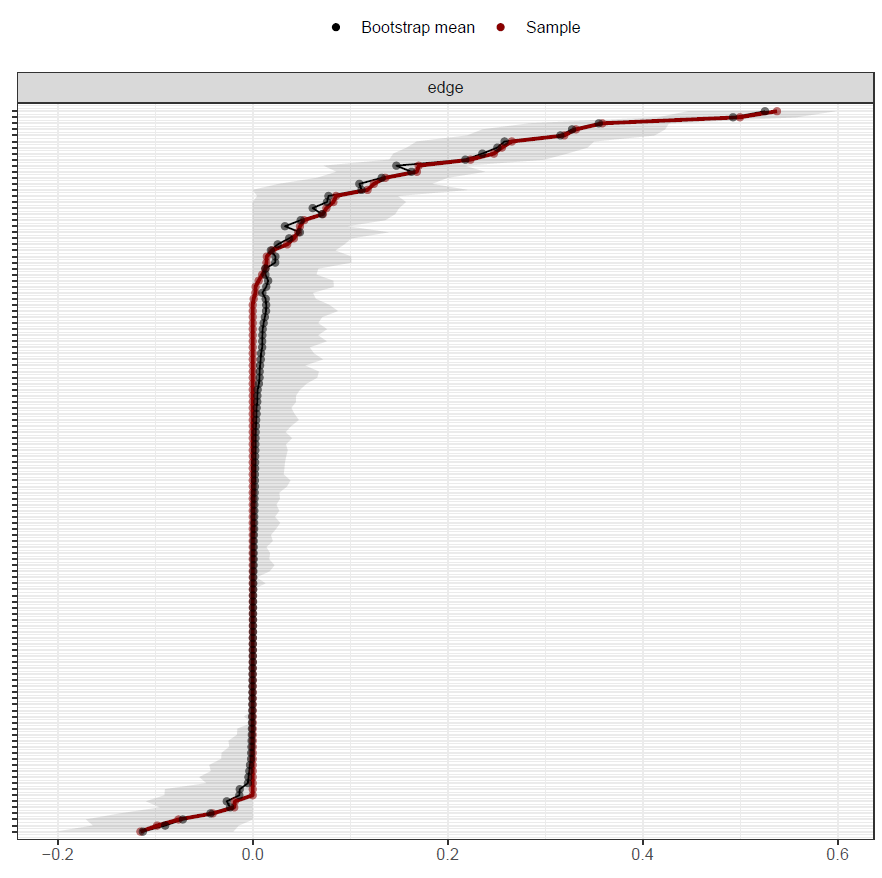


The accuracy of the network edges by non-parametric bootstrapping The grey area represents the bootstrap 95% confidence interval.
